# Somatosensory Disturbance and Long‐Term Exposure to Methylmercury: A Comprehensive Review

**DOI:** 10.1002/jat.70256

**Published:** 2026-05-29

**Authors:** Leonardo Breno do Nascimento de Aviz, Carolina Fonte de Aviz, Fabiana Costa Cardoso, Laura dos Santos Barros, Raphaely Cristiny Sanches Progênio, Mariângela Moreno Domingues, José Maria Farah Costa Júnior, Saul Rassy Carneiro, Carlos Araújo da Costa, Maria da Conceição Nascimento Pinheiro

**Affiliations:** ^1^ Tropical Medicine Center Federal University of Pará Belém Brazil; ^2^ Federal University of Pará Belém Brazil

**Keywords:** exposure, mercury, methylmercury, sensory disturbance

## Abstract

Chronic exposure to methylmercury remains a significant public health concern, particularly in populations with high fish consumption. Somatosensory disturbances are among the most frequently reported neurological manifestations, even at exposure levels below internationally established safety thresholds. This review aims to synthesize current evidence on somatosensory disturbances associated with long‐term methylmercury exposure in human populations. This comprehensive review findings from human studies investigating clinical, psychophysical, and neurophysiological aspects of somatosensory function in populations chronically exposed to methylmercury. Relevant literature was identified through searches in major scientific databases, and studies were selected based on their relevance to the topic. The reviewed studies consistently report distal, symmetrical somatosensory impairments, commonly described as a glove‐and‐stocking pattern. Psychophysical assessments demonstrate altered tactile, vibration, and thermal perception, while neurophysiological investigations reveal cortical and peripheral sensory dysfunctions. The evidence indicates that chronic methylmercury exposure is associated with clinically relevant somatosensory disturbances. These findings highlight the importance of incorporating somatosensory assessment into the clinical evaluation and surveillance of populations at risk.

## Abstract 2

Chronic exposure to methylmercury is associated with persistent somatosensory disturbances, even at levels below established safety thresholds. This review synthesizes evidence from human studies, highlighting the prevalence of sensory impairment and alterations in tactile, thermal, and vibratory perception. Both clinical and neurophysiological findings suggest central and peripheral nervous system involvement, reinforcing the importance of somatosensory assessment in populations exposed to methylmercury.

## 1 Background

Methylmercury (MeHg) is recognized as a potent toxin that can contaminate food, such as fish and seafood (de Almeida Ribeiro Carvalho et al. 2022). Other foods, such as rice (Feng et al. 2008) and wheat (Bakir et al. 1980), have been involved in environmental contamination, some poisoning large numbers of people, resulting in lasting neurological damage and even death.

One of the most important poisoning episodes involving food contaminated with organomercury compounds occurred in the 1950s in Minamata, Japan, which resulted in deaths as well as serious, irreversible neurological manifestations, known as Minamata disease (MD). In the 1960s, a similar episode, albeit with less intensity, occurred in Niigata, Japan (Maruyama et al. 2012). In 1970, an environmental accident occurred in Iraq resulting from the intake of bread made from wheat treated with a fungicide containing mercury (Hg), with serious effects on the central nervous system in adults and children (Maruyama et al. 2012). In these accidents, exposure was confirmed by concentrations of total mercury (HgT) or MeHg in blood or hair samples. The concentration of MeHg in hair is 250 times greater than that found in blood and has been used to determine exposure through food (World Health Organization (WHO) 1990).

The Hg, especially in the form of MeHg, is highly neurotoxic. It can accumulate in the body over time, leading to clinical manifestations that affect the central and peripheral nervous systems. These manifestations result in motor, cognitive, and sensory deficits. One of the first symptoms of Hg poisoning is sensory disturbance, which is characterized by paresthesia or dysesthesia in the extremities and can occur even in mild cases (Khoury et al. 2015). Somatosensory alterations can include peripheral damage to somatic sensory nerves, which can modify the threshold of tactile, pain, thermal, and vibratory perception; two‐point discrimination; and sensory reaction time (Khoury et al. 2013).

The combination of these symptoms may be characteristic, but each individual neurological symptom is not specific to organic Hg poisoning. Thus, a history of exposure to MeHg, a neurological examination, and the measurement of MeHg in the blood or hair are important to the diagnosis of MD (de Almeida Ribeiro Carvalho et al. 2022; WHO 1990).

Little is known regarding the effects of long‐term exposure to low doses of MeHg in adults. Studies on the long‐term neurotoxicological effects of MeHg exposure among consumers of fish predict that 5% of adults with Hg levels of 50 μg/g experience paresthesia (WHO 1990). However, more recent studies have suggested that MeHg can affect adults at levels lower than 50 μg/g (Maruyama et al. 2012; Harada et al. 1998; Auger et al. 2005).

Based on evidence of sensory disturbance in acute and severe forms of Hg poisoning in Niigata and Iraq, the World Health Organization (WHO) established 50 μg/g as the threshold level in hair for adult exposure to Hg (World Health Organization (WHO) 1990). As sensory disturbance is an important sign in the diagnosis of the acute and chronic forms of Hg poisoning and there is no definition on the lowest level of Hg for the appearance of somatosensory disorder, the aim of the present paper was to review specific primary studies evaluating sensory disorder in populations with long‐term exposure to MeHg through fish consumption, considering the frequency and intensity of the disturbance and the methods used for diagnosis.

## 2 Materials and Methods

This study is a comprehensive review aimed at synthesizing evidence on somatosensory disturbances associated with long‐term exposure to MeHg. The literature was explored using major scientific databases, including PubMed, CAPES Periodical Portal, Medline (via BVS), SciELO, LILACS, and IBECS focusing on human studies published between 1976 and 2025. Searches were conducted using combinations of keywords related to MeHg exposure and somatosensory function, such as “methylmercury,” “mercury exposure,” “somatosensory disturbance,” “sensory impairment,” “neuropathy,” and “human studies.”

Studies were selected based on their relevance to the topic, with emphasis on clinical, epidemiological, psychophysical, and neurophysiological findings related to somatosensory function. Due to the narrative nature of this review, no formal systematic selection protocol was applied.

Eligible publications included original research articles involving human populations chronically exposed to MeHg through environmental or dietary sources. Studies were selected based on their relevance to the objectives of this review, with particular emphasis on investigations reporting clinical manifestations, psychophysical assessments, and neurophysiological outcomes related to somatosensory function. Experimental animal studies, acute intoxication reports, and articles not addressing somatosensory outcomes were excluded.

Given the narrative nature of this review, no formal systematic selection protocol, quality scoring, or meta‐analytical procedures were applied. Instead, the selected studies were critically analyzed and thematically organized to provide an integrated overview of the current evidence on somatosensory disturbances associated with chronic MeHg exposure.

### 2.1 Clinical and Epidemiological Studies on Prolonged Exposure

In clinical epidemiological studies of long‐term exposure to MeHg, sensory disturbance was consistently observed, although its prevalence exhibited variability. In the majority of studies, sensory disturbances were found to manifest primarily as a reduced sense of touch in the extremities (as if wearing gloves and socks) and around the mouth. According to Bakir et al. (1980), the sensory disturbance in the Iraq tragedy was characterized by a variety of symptoms, including a tingling sensation, numbness, and the sensation of insects moving beneath the skin. On occasion, a burning sensation was also reported, with an asymmetrical distribution (Bakir et al. 1980).

The phenomenon is characterized by a loss of sensation, also referred to as hypoesthesia, or an increase in sensation, known as hyperesthesia. These sensations may be experienced in response to touch or pain. These alterations may manifest in isolated regions, asymmetrically, or in a spinal pattern, predominantly affecting the lower half of the body (lower limbs and the trunk below the waist). In more severe cases, sensory alterations may become diffuse, affecting all parts of the body (Takaoka et al. 2023) (Figure 1).

**FIGURE 1:**
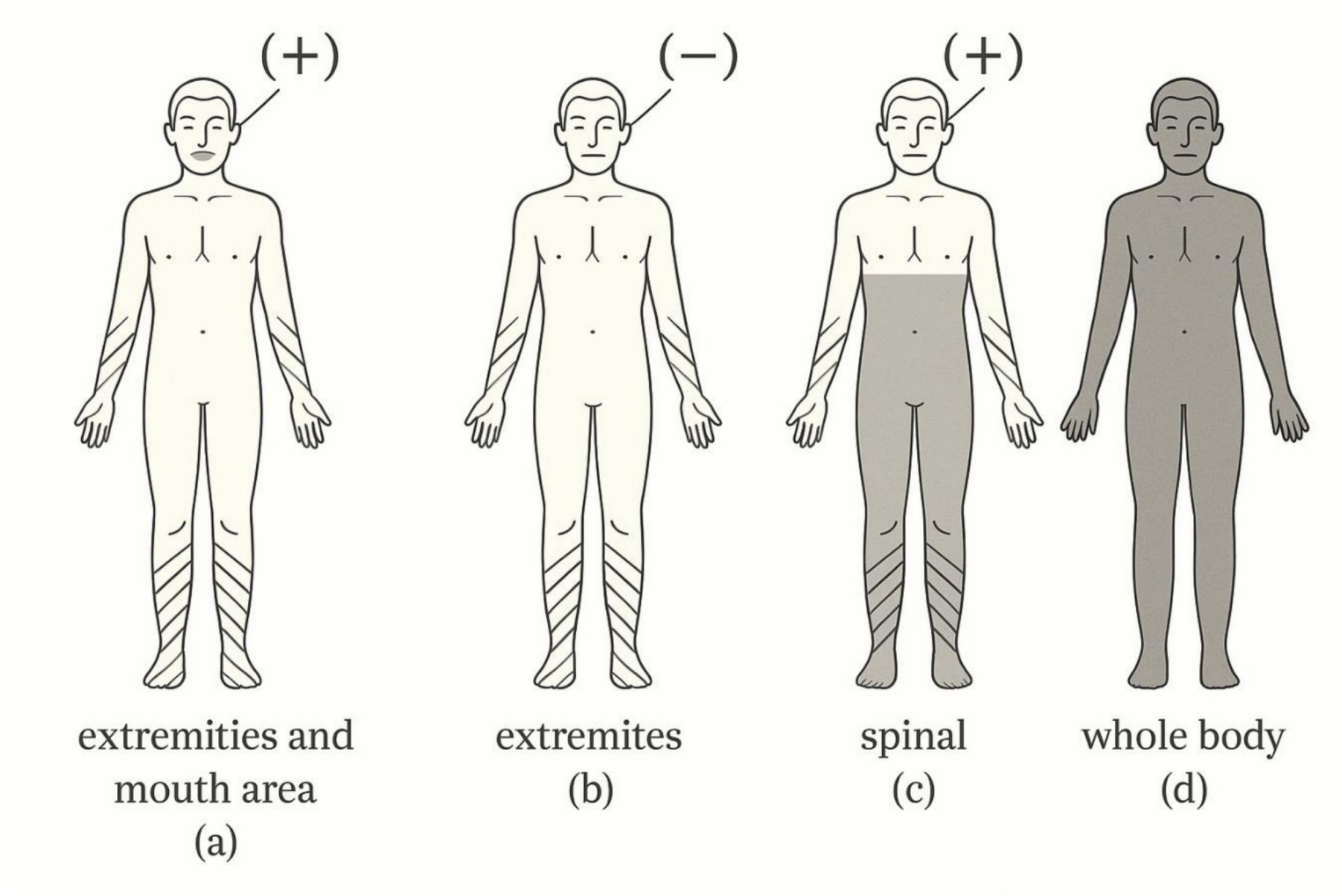
Varieties of sensory disturbance following exposure to MeHg. (a) Hyperesthesia in the extremities and perioral region. (b) Hypoesthesia restricted to the extremities (Bakir et al. 1980); a spinal pattern involving the lower half of the body (Maruyama et al. 2012); generalized distribution, with involvement of the entire body.

In Minamata and surrounding areas, the prevalence of somatosensory disturbances in epidemiological studies ranged from 25% to 100% (Harada et al. 1998; Uchino et al. 1995; Yorifuji et al. 2008; Yorifuji et al. 2009; Nakamura et al. 2014). In Niigata, where the environmental accident occurred with less intensity, the prevalence of sensory disturbances was very high (95.5%) (Maruyama et al. 2012).

Ninomiya et al. (1995) studied the effects of long‐term exposure to MeHg in individuals 10 years after the end of MeHg dispersion. The authors found significant neurological signs characteristic of MeHg poisoning, the highest frequency of which was hypoesthesia (73%) compared with the 20% rate found among individuals in a non‐polluted fishing village. Findings suggestive of sensory disturbance were found in 80.5%, with the “glove and stocking” type found in 42.9% (Ninomiya et al. 1995).

Uchino et al. (1995) investigated neurological findings in 77 patients with MD, as certified by autopsies performed between 1976 and 1994, and compared these findings with those of patients with classic MD in the follow‐up from 1957 to 1960. Patients previously with classic MD had sensory disturbance (100%) even 20 years following the onset of the disease. In both studies, Ninomiya et al. (1995) and Uchino et al. (1995), sensory disturbance remained years after the source of exposure was removed, suggesting that the effects of prolonged exposure may remain even though Hg levels are low.

Although high levels of Hg‐T together with the presence of sensory disturbance are important to the diagnosis of Hg poisoning, Harada et al. (1998) found that the disease appeared as mild, atypical “glove and stocking” MD (69.1%) and other neurological signs in many residents from contaminated areas in Japan and that hair Hg level lower than 10 ppm was not necessarily useful as a criterion for diagnosing chronic MD due to the long lapse of time, demonstrating how difficult it is to investigate the present extent of past Hg pollution (Harada et al. 1998).

On the other hand, Uchino et al. (2005) evaluated the temporal profile of the MD severity in certified patients using multiple logistic analysis and found that the disease is severest during the period of exposure to MeHg and is alleviated with time after the source of exposure is removed. When the aggravation of neurological symptoms occurs, the influences of complications may be substantial, suggesting that the differentiation of chronic MD due to MeHg based only on sensory disturbance is unviable, especially in older patients (Uchino et al. 2005).

In a large‐scale study conducted in three different areas of the Kumamoto Province in Japan, the population in two of which had been exposed for more than 10 years, the analysis of neurological manifestations related to the intensity of exposure to Hg (high, medium and low) revealed that sensory disturbances (extremities and around the mouth) were more prevalent in highly exposed area, but the prevalence in the area of medium exposure was also higher than in the control area (Yorifuji et al. 2008).

Although dose–response relationships were not clear for some neurological signs in the internal comparison, a clear dose–response relationship was found between hair Hg content and sensory loss around the mouth. This neurological sign was substantially increased in the exposure categories of > 20 to 50 μg/g, which are lower than the current WHO limit for Hg exposure (Yorifuji et al. 2009).

Among victims of MeHg poisoning in Niigata who had hair Hg concentrations less than 50 μg/g, bilateral sensory disturbance in the distal extremities was the most frequent finding. Therefore, levels below the current WHO limits (50 μg/g of hair) can cause neurological signs, particularly sensory disturbance (Maruyama et al. 2012; World Health Organization (WHO) 1990).

The association between exposure to MeHg through the consumption of whale, which is a common habit in some areas of the globe, and neurological disorders was evaluated in adults in the city of Taiji, Japan. The geometric mean Hg concentration of 194 individuals was 14.9 μg/g. Twelve individuals had Hg levels higher than 50 μg/g, with no cases “glove and stocking” sensory disturbance. In other individuals with Hg lower than 50 μg/g, three of neurological signs were found but were associated to causes other than MeHg exposure (Nakamura et al. 2014). The main epidemiological findings from studies conducted in Japan are summarized in Table 1.

**TABLE 1:** Sensory disturbance in individuals with long‐term exposure to MeHg: Epidemiological studies in polluted areas of Japan from 1995 to 2024.

Sensory disturbance	Location	Prevalence	HgT level μg/g	Authors
Sensory disturbance “glove and stocking” type	Minamata, Japan	80.5 42.9%	_	Uchino et al. (1995)
Sensory disturbance (“glove and stocking” type)	Japan Polluted area Non‐polluted area	73% 20%	35 ± 20	Ninomiya et al. (1995)
Sensory disturbance “glove and stocking” type	Japan	75% 69.1%	< 10	Harada et al. (1998)
Sensory disturbance (“glove and stocking” type)	Minamata, Japan With/without paresthesia	100%	Mean: 2.4 ± 1.9	Uchino et al. (2005)
Sensory disturbance (“glove and stocking” type)	Minamata Goshonoura Kumamoto	31% 5.7% 4.4%	30 (high exposure) 21.5 (medium exposure) 2.1(low exposure)	Yorifuji et al. (2008)
Bilateral sensory disturbance	Japan	23% 25% 39% 58%	0–10 > 10 to 20 > 20 to 50 > 50	Yorifuji et al. (2009)
Sensory disturbance (“glove and stocking” type)	Niigata, Japan	95.8%	< 50	Maruyama et al. (2012)
Sensory disturbance (“glove and stocking” type)	Taiji, Japan (whale meat consumption)	3/182 1/12	< 50 > 50	Nakamura et al. (2014)
Sensory disturbance	Minamata, Japan	94.44%	_	Takaoka et al. (2023)
Sensory disturbance	Miyanokawachi, Japan Himedo, Japan Nagashima, Japan Amami, Japan	58.6% 53.9% 37.8% 1.4%	_	Takaoka et al. (2024)

In the study conducted by Takaoka et al. (2023), 197 individuals chronically exposed to MeHg from the Minamata region were analyzed and compared with 130 unexposed controls. The investigative approach entailed the utilization of psychophysical assessment instruments to identify somatosensory variables. The aforementioned variables encompassed of assessments, including tactile sensitivity evaluated through Semmes–Weinstein monofilaments, two‐point discrimination tests, deep sensitivity measured using 128‐Hz tuning forks, and proprioception. The results indicated a higher prevalence of deficits in tactile and pain sensitivity, along with alterations in deep sensitivity and two‐point discrimination (Takaoka et al. 2023).

Furthermore, Takaoka et al. (2024) conducted studies in populations from coastal areas far from Minamata City that were not traditionally recognized as contaminated by MeHg. These areas included the districts of Miyanokawachi, Himedo, and Nagashima. The researchers compared these populations with the control population of Amami Island. The evaluation included structured questionnaires, standardized neurological examinations, and psychophysical tests such as Semmes–Weinstein monofilaments and tuning fork vibration assessment.

The authors identified sensory disturbances, primarily in the extremities involving touch and pain, with patterns consistent with chronic exposure to MeHg through seafood consumption. While the exact rates of environmental exposure were not directly measured, the results revealed a significant number of participants exhibiting clinical signs consistent with sensory intoxication, even in regions distant from historically recognized zones of contamination (Takaoka et al. 2024).

Studies conducted in populations outside Japan are summarized in Table 2. Populations in other countries subjected to prolonged exposure to Hg through diet have varied rates of somatosensory disturbances with low Hg concentrations in hair samples. In a study involving the aboriginal Cree in Quebec, Canada, the prevalence was 5.3% (Auger et al. 2005). In Serra Pelada, Brazil, the prevalence of paresthesia was 64.93% (Corbett et al. 2007). In all studies, somatosensory disturbance was associated with Hg concentrations lower than 50 μg/g. The “glove and stocking” type sensorial disturbance was found 14% of a river community in the Amazon (Harada et al. 2001) and 54.4% on an aboriginal reservation in Canada (Harada et al. 2005).

**TABLE 2:** Sensory disturbance in long‐term exposure to MeHg: Epidemiological studies in polluted in areas outside Japan from 1994 to 2014.

Sensory disturbance/location	Prevalence	HgT level μg/g	Authors
Sensory disturbance Amazon river community, Brazil	32% 14% (“glove and stocking” type)	> 20	Harada et al. (2001)
Sensorial disturbance Aboriginal Cree, Quebec, Canada	5.3%	< 50	Auger et al. (2005)
Sensory disturbance Aboriginal reservation, Canada	54.4% (“glove and stocking” type) 15.8% (around mouth)	< 10	Harada et al. (2005)
Sensory disturbance Serra Pelada, Brazil	64.9%	< 10	Corbett et al. (2007)
Sensory disturbance Tapajos river, Brazil	13.3% (two‐point discrimination)	Mean: 9.19 ± 6.4	Khoury et al. (2013)
Sensory disturbance Amazon River community, Brazil	“Glove and stocking” type Two‐point discrimination	8.8 ± 8.5	Khoury et al. (2015)
Sensory disturbance Indigenous community, Suriname	59% (“glove and stocking” type) 31.8% (two‐point discrimination)	20 (2008) 14 (2012)	Peplow and Augustine (2014)
Aboriginal community, Canada Japan	Touch disturbance Elderly: 59% Exposed: 83% Pain disturbance Elderly: 59% Exposed: 97%	—	Takaoka et al. (2014)

The common threads in these studies were exposure to Hg from freshwater fishes, prolonged exposure, and low Hg levels in hair samples. Studies on somatosensory disorder associated with prolonged exposure to Hg conducted in Brazil are restricted to the Amazon River communities, particularly in areas near centers of gold prospecting activities in the state of Pará with a history of weekly fish intake.

In a Canadian study on an aboriginal community living in areas contaminated with Hg exposed to low concentrations of Hg through fish consumption, Harada et al. (2005) found a 54.4% prevalence rate of “glove and stocking” sensory disorder and 15.8% rate around the mouth (Harada et al. 2005). In another Canadian study, the prevalence ranged from 0 to less than 6% in cross‐sectional and controlled studies, respectively (Auger et al. 2005).

However, Takaoka et al. (2014) found a high prevalence rate using semiquantitative methods in a study on sensory disturbance comparing residents exposed to MeHg in the Minamata district of Japan, control residents from a non‐polluted area in Japan, and patients exhibiting symptoms of Hg poisoning in northwestern Ontario, Canada (Takaoka et al. 2014). In an indigenous community in Suriname residing near a gold mining area that consumed fish, “glove and stocking” sensory disturbance was the most frequent neurological manifestation of exposure to Hg, occurring in 13 of 22 patients evaluated in 2012, with Hg exposure levels of 14 μg (Peplow and Augustine 2014).

Clinical evaluations were performed on 235 residents near gold mines in Serra Pelada, Brazil, exposed to Hg through the consumption of fish, with 64.93% reporting symptoms of somatosensory disturbances and 24.66% reporting other neurological signs (Corbett et al. 2007). Although these neurological changes were not directly related to MeHg and could have resulted from other conditions, such as alcoholism, trauma, diabetes, and exposure to pesticides, Hg exposure may also have contributed. At this time of the study, the gold mining activities had been suspended and the population consumed contaminated fish.

More recently, Khoury et al. (2013) conducted a study in the Amazon region to assess the neurological effects of long‐term exposure in a Tapajós River community comparing populations in different river basins and considering different exposure levels.

The authors found a low frequency of sensory alterations based on conventional neurological testing, despite Hg exposure levels. However, when psychophysical tests were employed, significant sensory loss was found. The authors performed tactile sensation, vibration sensation, and two‐point discrimination (smallest distance at which two points are perceived as two separate contacts) tests and all exposed individuals had higher thresholds than control subjects (Khoury et al. 2013). The authors concluded that long‐term exposure to Hg may have been the etiological agent of the psychophysical somatosensory losses found in this population (Khoury et al. 2015).

In populations exposed to Hg, sensory disturbance was investigated using psychophysical tests in clinical studies conducted in Minamata, Japan, Canada, and the Brazilian Amazon to quantify these manifestations. Quantitative assessments of somatosensory function are presented in Table 3.

**TABLE 3:** Quantitative assessment of somatosensory function.

Location	Pain threshold	Tactile sensation threshold	Duration of vibration sensation	Two‐point discrimination	Authors
Minamata, Japan.	Altered (+)	NI	NI	NI	Yoshida et al. (1992)
Minamata, Japan (surrounding areas)	NI	Altered (+)	NI	NI	Takaoka et al. (2004)
Minamata, Japan	NI	Altered (+)	NI	Altered (+)	Ninomiya et al. (2005)
Minamata, Japan	NI	Altered (+)	Altered (−)	Altered (+)	Takaoka et al. (2008)
Northwestern Ontario, Canada	Altered (+)	Altered (+)	Altered (−)	Altered (+)	Takaoka et al. (2014)
Tapajos, Amazon region, Brazil	NI	Altered (+)	Altered (−)	Altered (+)	Khoury et al. (2013)

*Note:* (+) increased threshold (reduced sensitivity); (−) decreased threshold.

Abbreviations: NA, not available; NI, not investigated.

Using an algesimeter, which measures sensitivity to pain (heat stimulus), Yoshida et al. (1992) found that the pain threshold was increased in the extremities and body of individuals with MD (Yoshida et al. 1992). Takaoka et al. (2014) also found a higher frequency of an increased pain threshold in Canadian aborigines (59%) and elderly Japanese (50%) exposed to Hg when compared with controls (Takaoka et al. 2014).

Takaoka et al. (2004) employed abrasive aluminum‐oxide paper of different grits as a stimulus in a psychophysical sensory examination of fine‐surface‐texture discrimination and somatosensory loss in individuals with and without numbness. “Glove and stocking” sensory disturbance was found in 100% of the elderly group (60 to 79 years of age) with complaints paresthesia and who had been exposed to Hg for decades Minamata, Japan, with present hair Hg levels of 2.4 ± 1.9 μg/g. The two‐point discrimination test revealed an increased threshold in the thumbs, tongue, forefingers, and lips of individuals with MD. According the authors, the results suggest that the persistent somatosensory disorders in such patients were probably not caused by injury to peripheral nerves (Takaoka et al. 2004).

Ninomiya et al. (2005) investigated touch thresholds measured using the Semmes–Weinstein pressure esthesiometer (a set of modified filaments) and two‐point discrimination test using an adjustable compass. The authors found that touch thresholds were significantly increased in comparison with those of the control group. The two‐point discrimination thresholds were increased on both forefingers and the lip in both groups. These changes were observed in patients with MD with complaints of paresthesia in the distal parts of the extremities and around the lips even 30 years after exposure had ceased, demonstrating the persistence of sensory disturbance and suggesting an association with the function of the cerebral cortex (Ninomiya et al. 2005).

Using quantitative test, Takaoka et al. (2008) evaluated a cohort of 197 residents from the Minamata area with a high frequency of sensory disturbance who had a history of fish consumption during the polluted period. The authors found signs and symptoms consistent with MeHg exposure that could be diagnosed even in the presence of other aging‐related symptoms and neurological complications. Tactile sensation thresholds of the chest were higher than those for the index finger and thumb; the authors also found impairment regarding the sense of vibration and higher two‐point discrimination thresholds in individuals with a history of exposure to MeHg (Takaoka et al. 2008). Ninomiya et al. (2005) found similar impairments in the distal limbs, proximal limbs, and trunk in individuals exposed to Hg (Ninomiya et al. 2005).

In a community of an area exposed to Hg in the Tapajós River basin in the Brazilian Amazon, Khoury et al. (2015) investigated the tactile sensation threshold using the Semmes–Weinstein kit (20 monofilaments of different diameters), vibration sensation using a tuning fork (128 Hz), and two‐point discrimination using a two‐point discriminator. The authors found impaired tactile sensation, sense of vibration, and two‐point discrimination in comparison with the control community (Khoury et al. 2015). Impaired somatosensory aspects were also found in populations from Japan. Takaoka et al. (2008) and Ninomiya et al. (2005) found that the two‐point discrimination thresholds were higher than those found in the study by Khoury et al. (2015). This difference may be attributed to the level of Hg exposure and age of the subjects. Although current exposure to Hg in Japan is similar to that found in the Amazon region, the Japanese individuals studied were exposed to much higher levels in the past. Moreover, the individuals in the Amazon were generally younger than those in the Japanese studies (Khoury et al. 2015; Ninomiya et al. 2005; Takaoka et al. 2008). According to Uchino et al. (2005), one must bear in mind the effects of an advanced age on sensory disturbance in individuals with prolonged exposure to Hg, making it more difficult to diagnose MeHg poisoning in such cases (Uchino et al. 2005).

### 2.2 Electrophysiological and Pathological Studies

The damage site has been discussed for a long time, and early studies reported that sensory disturbance was not caused by injury to peripheral nerves; this idea was based on electrophysiological and morphological findings (Eto et al. 2002). Patients with chronic poisoning reported sensory reduction in the distal extremities in the pattern denominated “glove and stocking” distribution, which is a characteristic feature of peripheral neuropathy when examined using conventional sensory examinations with a paint brush and pinprick. Tokuomi et al. (1982) investigated short‐latency somatosensory evoked potentials in patients with MD more than 10 years after the cessation of exposure to MeHg and concluded that the damage causing persistent sensory disturbance in such patients is in the somatosensory cortex (Tokuomi et al. 1982).

A histopathological study revealed that both the sensory cortex (postcentral gyrus) and peripheral sensory nerves were impaired. In autopsies of cases of MD, it became apparent that the brain damage was not restricted to the calcarine cortex, but was relatively widespread. Less severe lesions, believed to be responsible for the motor symptoms in patients with MD, were often found in the precentral, postcentral, and lateral temporal cortices (Figure 2).

**FIGURE 2:**
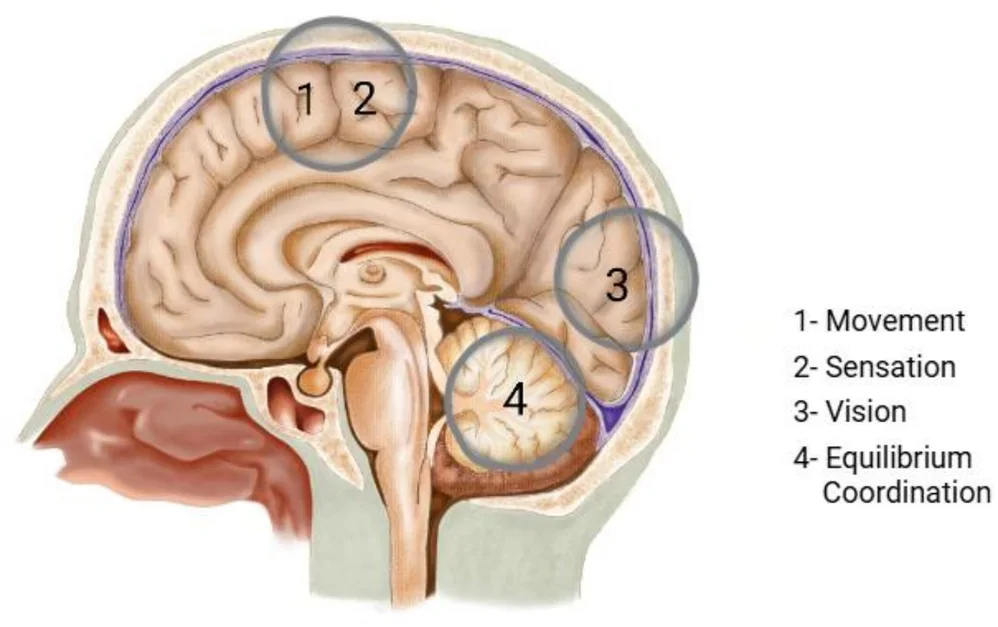
Specific area that may be affected by chronic exposure to MeHg. Primary motor cortex area (de Almeida Ribeiro Carvalho et al. 2022). Primary somatosensory cortex area (Feng et al. 2008). Occipital lobe area (Bakir et al. 1980). Cerebellum area (Maruyama et al. 2012).

In a neurophysiological study by Nakamura et al. (2023), magnetoencephalography was employed to evaluate somatosensory cortical responses in individuals with chronic MD. Compared with healthy controls, the study found that patients exhibited decreased amplitude of the N20m component, increased P20m amplitude, and a higher rate of absent cortical responses to median nerve stimulation. These results suggest that functional abnormalities persist in the primary somatosensory cortex, even years after exposure has ceased (Nakamura et al. 2023).

Biopsy and autopsy findings of peripheral nerves in fisherman with MD revealed endomysial fibrosis and electron microscopy of the sural nerve revealed regenerated myelin sheaths, suggesting peripheral nerve degeneration in patients with MD due to the toxic injury caused by exposure to MeHg (Figure 3) (Eto et al. 2002).

**FIGURE 3:**
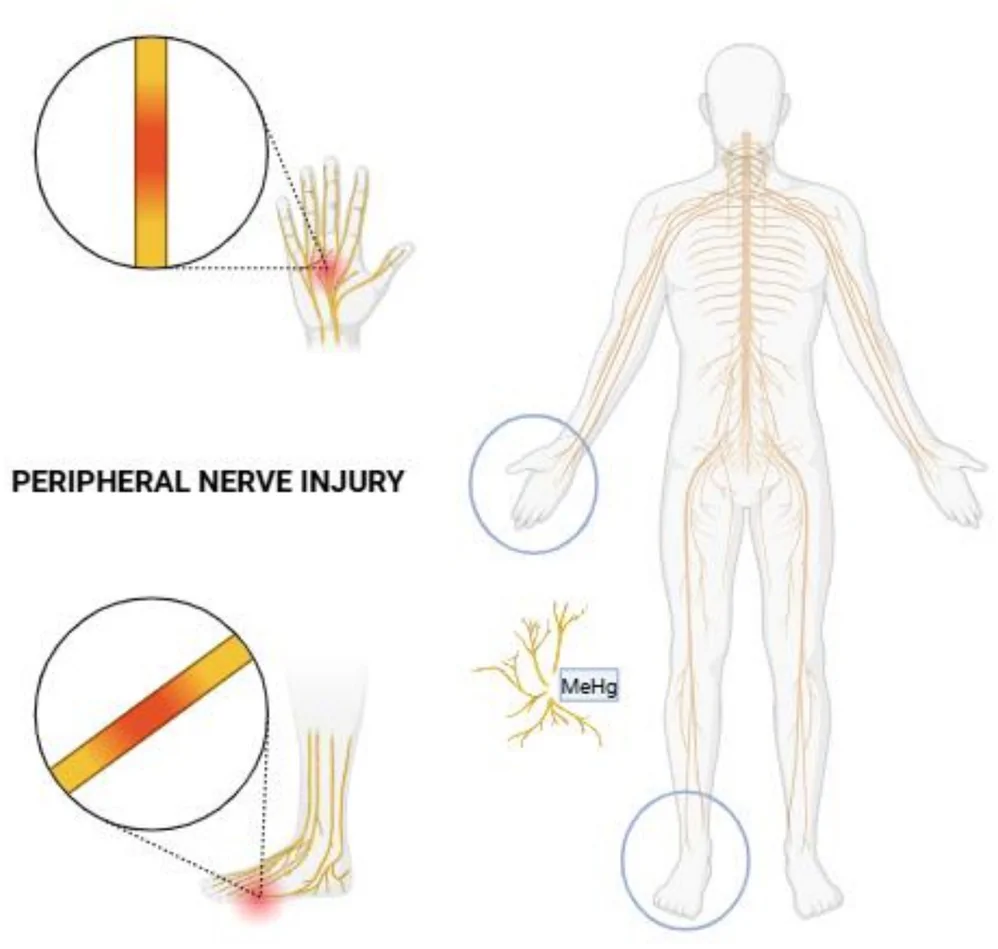
Schematic representation of peripheral nerve injury associated with chronic MeHg exposure.

## 3 Conclusion

Therefore, in the current literature, few studies conducted in areas exposed to MeHg exposed have considered sensory disturbance, despite being the earliest and the most prevalent symptom of MD. This subjective symptom should be used to compliment conventional neurological examinations. Patients can confirm sensory abnormalities, especially in cases of prolonged exposure to low concentrations of Hg, thereby contributing to the diagnosis of Hg poisoning and the establishment of measures to be taken by medical authorities.

A history of prolonged exposure to MeHg through diet, the persistence of Hg concentration in hair samples above 10 μg/g, and the presence of somatosensory disturbance in a population with high fish intake are fundamental signs for the diagnosis of MeHg poisoning. Particularly in regions where exposure levels are known, the investigation of somatosensory disturbance should be a routine medical practice. Moreover, knowledge on Hg levels in the fish consumed and educational practices addressing the risk of poisoning are important measures to be taken by health authorities to prevent poisoning and control exposure to MeHg in areas of risk.

## Funding

This study was supported by the Pró‐reitoria de pesquisa e pós‐graduação—Universidade Federal do Pará (UFPA) and Coordination for the Improvement of Higher Education Personnel (CAPES), Brazil.

## Conflicts of Interest

The authors declare no conflicts of interest.

## Data Availability

The data that support the findings of this study are available from the corresponding author upon reasonable request.
